# Why evolutionary biologists should get seriously involved in ecological monitoring and applied biodiversity assessment programs

**DOI:** 10.1111/eva.12215

**Published:** 2014-10-29

**Authors:** Jakob Brodersen, Ole Seehausen

**Affiliations:** 1 Department of Fish Ecology and Evolution, EAWAG Swiss Federal Institute of Aquatic Science and Technology, Center for Ecology, Evolution and BiogeochemistryKastanienbaum, Switzerland; 2 Division of Aquatic Ecology and Evolution, Institute of Ecology and Evolution, University of BernBern, Switzerland

**Keywords:** conservation, ecosystem monitoring, evolutionary biology, genotypes, management, phenotypes

## Abstract

While ecological monitoring and biodiversity assessment programs are widely implemented and relatively well developed to survey and monitor the structure and dynamics of populations and communities in many ecosystems, quantitative assessment and monitoring of genetic and phenotypic diversity that is important to understand evolutionary dynamics is only rarely integrated. As a consequence, monitoring programs often fail to detect changes in these key components of biodiversity until after major loss of diversity has occurred. The extensive efforts in ecological monitoring have generated large data sets of unique value to macro-scale and long-term ecological research, but the insights gained from such data sets could be multiplied by the inclusion of evolutionary biological approaches. We argue that the lack of process-based evolutionary thinking in ecological monitoring means a significant loss of opportunity for research and conservation. Assessment of genetic and phenotypic variation within and between species needs to be fully integrated to safeguard biodiversity and the ecological and evolutionary dynamics in natural ecosystems. We illustrate our case with examples from fishes and conclude with examples of ongoing monitoring programs and provide suggestions on how to improve future quantitative diversity surveys.

## Introduction

### Biodiversity assessment, monitoring, and research

The realization of the necessity to integrate past and present ecological processes across multiple spatial scales (Ricklefs and Schluter 1993) has transformed community ecology and has become central for the design of many ecosystem assessment, monitoring, and management programs (Swetnam et al. 1999). In return, several ecosystem assessment and monitoring programs are now collecting large data sets that allow ecologists to test predictions of ecological theory (e.g., Jeppesen et al. 2005). Despite the increasing realization that evolution happens at the same time scales (Hendry and Kinnison 1999; Hendry et al. 2007), no such productive interactions have developed between ecosystem monitoring and evolutionary biology. Here, we argue that this is both unwarranted and problematic from a point of view of both science and conservation.

With this review, we aim to promote the integration of evolutionary biology thinking into existing ecological monitoring and applied biodiversity assessment programs. In our view, this requires both a larger involvement of evolutionary biologists in existing monitoring and assessment programs, but also a larger understanding of managers of the importance of often contemporary evolutionary processes in ecosystem structure and dynamics. We do so by first introducing the need for assessment of genetic and phenotypic diversity within species and populations with specific sections on how contemporary evolution can change biodiversity and why the assessment of intraspecific variation is indispensable. We then turn our attention to the most common problems associated with ignoring evolution in monitoring programs, before describing the importance of historical collections. Finally, we focus on large-scale assessment of ecosystems and biodiversity and suggest strategies for future monitoring programs.

We here consider biodiversity survey and monitoring, that is, repeated assessment, together. Data collected in survey programs are in many cases not repeated temporally, but still have high value for conservation, management, and advancing biological theory. For example, in the Swedish National Registry of Survey test-fishing (NORS: http://www.slu.se/en/departments/aquatic-resources/databases/national-register-of-survey-test-fishing-nors/), 2072 (63%) of the 3283 lakes in the database have only been surveyed on a single occasion (Kinnerbäck 2013), but still provide valuable data for estimation of, for example, biogeography (also see section on Large-scale assessment of ecosystems and biodiversity). In this paper, we will use the words assessment and monitoring in their widest sense.

### The need for assessing genetic and phenotypic diversity within species and populations

Biodiversity continues to decline globally (Butchart et al. 2010; Pereira et al. 2010), with serious consequences for ecosystem structure and functioning (Cardinale et al. 2006; Duffy et al. 2007; Hooper et al. 2012), as well as for the services provided by ecosystems (e.g., Worm et al. 2006; Cardinale et al. 2012). To effectively work against this trend, it is crucial to realize that biodiversity is a dynamic outcome of the interaction of past and ongoing ecological, demographic, and evolutionary processes. Changing environments may trigger either primarily demographic or primarily evolutionary responses in any individual population, and both types of responses may interact and feedback on each other (Post and Palkovacs 2009; Schoener 2011). At community and ecosystem levels, increasingly complex interactions between demography and evolution are expected, as multiple interacting species may change both demographically and through evolution. Finally, the interaction of both types of processes will govern responses of diversity at its different hierarchical levels in different spatial contexts, that is, alpha, beta, and gamma diversity for genotypes, phenotypes, populations, species, and higher taxonomic categories. Biological monitoring programs need to be able to uncover the true complexity of these dynamics and to eventually permit predicting biodiversity responses to alternative scenarios of future environmental change. Biodiversity surveys and monitoring should therefore, besides documenting the current state of ecosystem structure, species diversity, and its evolutionary history, permit documentation of diversity below the species level, and contemporary ecological and evolutionary processes. Besides direct benefits to ecosystem management (Hughes et al. 2008), such integrated data collection would generate significant benefits for advancements in ecology and evolutionary biology and their synthesis and the resulting feedbacks between fundamental research, monitoring, and management of biological diversity would perhaps facilitate the end of their traditional divorce.

Similar to how the integration of past and present ecological processes has transformed community ecology (Ricklefs and Schluter 1993) and ecosystem assessment, monitoring, and management programs (Swetnam et al. 1999), there is – at least in theory – a growing realization of the needs for integrating evolutionary process into modern monitoring concepts (Schwartz et al. 2007; Laikre et al. 2010; Hansen et al. 2012). Unfortunately, practical reality is very far from beginning to achieve this. Integrating evolutionary process requires genetic and phenotypic data for individuals within populations. This is important not only to document existing biodiversity below the species level, but also to obtain insight into ongoing and predict future processes at population level, and how these are affected by environmental change. The idea of integrating data for several different levels of biodiversity into ecological monitoring programs is not new (e.g., Noss 1990). However, whereas biodiversity was often seen as a product of past evolution that generated, and current ecological processes that sort diversity, we emphasize that biodiversity results from ecological and evolutionary processes that dynamically interact at any time scale. Biodiversity at its local (alpha) level, which is most frequently measured in monitoring programs, is not merely lost or gained, but may shift its composition through replacement of one species by another, through genetic replacement of populations or species through introgressive hybridization, and through the collapse of distinct populations and species into fewer. The first of these processes will be considered biodiversity-neutral at the alpha level, and the latter two will go unnoticed in standard monitoring of diversity at classical species level. However, all three types of shift will in most cases result in a loss of diversity at the larger spatial scale (beta, gamma). Such loss is particularly obvious when globally rare or endemic diversity units are replaced with globally common ones (Thuiller et al. 2011; Villéger et al. 2011). Such shifts may occur as replacements on all levels from genes to species (see examples below), which may in principle all have profound effects on the dynamics and functioning of local ecosystems (e.g., Schindler et al. 2010; Farkas et al. 2013). In the following, we discuss elements available and new elements needed for ‘evolution-aware’ monitoring of biodiversity.

Over recent years, the concept of genetic monitoring has received increased attention (e.g., Schwartz et al. 2007; Hansen et al. 2012). However, such genetic monitoring concepts have often been presented as an alternative to traditional ecological monitoring programs. We agree with the necessity of genetic monitoring, but argue that it ought to be considered one of the several elements that need to be integrated into evolution-aware assessment and monitoring, the others being classical ecological monitoring and monitoring of diversity below species level.

### Contemporary evolution can rapidly change biodiversity

**Figure 1:**
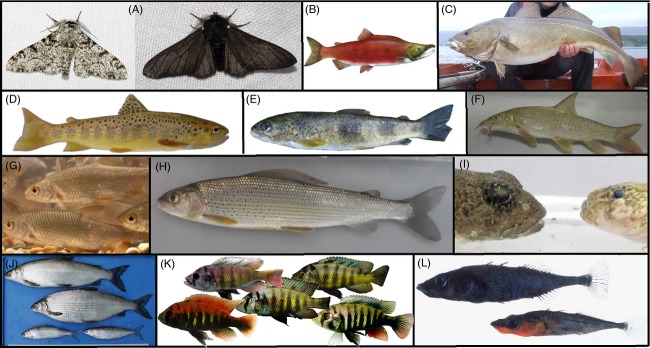
Overview of organisms mentioned in text: (A) light and dark phenotypes of peppered moth (*Biston betularia*), (B) sockeye salmon (*Oncorhynchus nerka*), (C) Atlantic cod (*Gadus morhua*), (D) Atlantic trout (*Salmo trutta*), (E) Rhône trout (*Salmo rhodanensis*), (F) barbel (*Barbus barbus*), (G) roach (*Rutilus rutilus*), (H) grayling (*Thymallus thymallus*), (I) two sympatric distinct phenotypes of sculpins (*Cottus* spp.) from Lake Thun, Switzerland, (J) whitefish species pair from Lake Walen, Switzerland (male and female *Coregonus duplex* (top) & *C. helingus* (bottom)), (K) phenotype gradient in a Cichlid species pair (*Pundamilia nyereri* and *P. pundamilia*) from Lake Victoria, (L) threespine stickleback species pair from Enos Lake, BC, Canada (*Gasterosteus* spp.). Photograph courtesy: (A) ‘Biston.betularia.7200’ and ‘Biston.betularia.f.carbonaria.7209’ by o.leillinger@web.de. Licensed under Creative Commons Attribution-Share Alike 3.0 via Wikimedia Commons – http://commons.wikimedia.org/wiki/File:Biston.betularia.7200.jpg#mediaviewer/File:Biston.betularia.7200.jpg & http://commons.wikimedia.org/wiki/File:Biston.betularia.f.carbonaria.7209.jpg#mediaviewer/File:Biston.betularia.f.carbonaria.7209.jpg, (B) ‘Oncorhynchus nerka’ by Timothy Knepp of the Fish and Wildlife Service. – US Fish and Wildlife Service. Licensed under Public domain via Wikimedia Commons – http://commons.wikimedia.org/wiki/File:Oncorhynchus_nerka.jpg#mediaviewer/File:Oncorhynchus_nerka.jpg, (L) Eric B. Taylor, University of British Columbia. All other photographs by the authors.

Traditionally, evolutionary and ecological processes were assumed to work on time scales that differed by orders of magnitude (Slobodkin 1961). Hence, observed diversity in nature was assumed to be a result of a relatively ancient evolutionary past that generated diversity and contemporary ecological processes that sort it (Carroll et al. 2007). Despite the long-standing realization that ecology is the major driver of natural selection, it was only in recent years that ecologists and evolutionary biologists began to realize that ecological process can drive evolutionary change at largely overlapping time scales (Hendry and Kinnison 1999; Hendry et al. 2007). Examples include the industrial melanism, that is, rapid change in phenotypes of the peppered moth, *Biston betularia* (Fig.1A), in response to human-induced change in selection environment (e.g., Kettlewell 1956) and the rapid evolution of reproductive isolation between beach and river spawning ecotypes of an introduced salmon population (Hendry et al. 2000; Fig.1B). This realization has important implications for nature conservancy and ecosystem management, but it has yet to be embraced by applied biodiversity monitoring. This is urgent because there is growing evidence that the increased rate of environmental change driven by human impact can speed up evolutionary processes (Hendry et al. 2008) including in ways that cause the rapid loss of biodiversity through evolution (Seehausen 2006). Adaptation and its loss, the reversal of speciation, and even incipient speciation can occur on contemporary time scales (Hendry et al. 2007; Seehausen et al. 2008; Abbott et al. 2013; Kleindorfer et al. 2014), population recovery can be facilitated or constrained by evolutionary processes (Lancaster et al. 2006), and biological invasions are often fueled by evolutionary change within the invasive populations (Kolbe et al. 2004; Allan and Pannell 2009; Lucek et al. 2013). Collectively, this suggests that evolutionary biology should be considered a central element in practical applications such as ecological monitoring (Thompson 1998; Jørgensen et al. 2007).

### Why the assessment of intraspecific variation is indispensable

Evolution is the engine that generates biological diversity, but individual variation is the fuel. We argue for a need of collecting data to describe distributions of individual variation both within and between species and populations for evolution-aware monitoring. By individual variation, we refer to genetic variation and trait variation but also variation in more highly dimensional phenotypes, that is, ecotypic variation. It is important that these data are fully integrated into biodiversity monitoring efforts to be able to follow changes in the distributions of phenotypes and genotypes through time (see subsequent sections).

We turn our attention to fisheries management for examples. Harvest of wild populations is commonplace in fisheries and often has substantial effects on the genotypic and phenotypic composition of the harvested species (e.g., Jørgensen et al. 2007; Allendorf et al. 2008; Heino and Dieckmann 2009). In many heavily harvested fish, populations have changed life histories, most often toward slower growth rates and earlier maturation (Olsen et al. 2004; Heino and Dieckmann 2008), and many local populations have gone extinct. North Atlantic cod (Fig.1C) once made for one of the largest commercial fisheries and has been managed as a single large stock until its dramatic collapse in the second half of the 20th century. In the course of the 2000s, it became apparent that the overfishing of North Atlantic cod led to the loss of large and unduly ignored biodiversity: North Atlantic cod turned out to be a complex of regionally diverse, genetically distinct, stocks with diverse ecological adaptations, several of which have undergone disproportionally large collapses as a consequence of overfishing (e.g., Hutchinson et al. 2003; Hilborn and Litzinger 2009). We suspect that had evolutionary biologists ever studied cod with the methods used to study cichlid fish or stickleback, for example, by detailed assessment and description of phenotypic diversity, its heritability and environmental correlates, they would have come to discover an adaptive radiation with several young but reproductively isolated species. Much of this is now lost.

When a traditional fisheries management concept of maximum sustainable yield is applied to a mixed-stock and mixed-species fishery, it results in a ratchet-like extirpation of the less productive species and populations (Allendorf et al. 2008), and as populations decline (be it due to exploitation, habitat loss e.g., due to eutrophication or other causes), individuals are expected to start breeding with individuals of other still abundant populations and species, thus triggering a cascade of irreversible and rapid genetic and phenotypic diversity loss (e.g., Vonlanthen et al. 2012). This way fisheries management practice and environmental change often lead to the collapse of previously differentiated stocks and species (e.g., Todd and Stedman 1989; Lancaster et al. 2006), a process referred to as ‘speciation reversal’ (Seehausen et al. 2008).

Such human-induced changes in the diversity and distribution of populations that differ in their adaptations, and may be reproductively isolated incipient species, are cases of evolution on ecological time scales when observed at local spatial scale and loss of biodiversity when observed at global scale, that may have consequences for ecosystem dynamics, structure, and services (Worm et al. 2006; Heino and Dieckmann 2009; Palkovacs et al. 2012). Importantly, such evolutionary responses to environmental change will not be quickly ameliorated by adjusting management schemes (Enberg et al. 2009) or restoring habitat. Some of the affected cod stocks have indeed shown little evidence of postcollapse recovery, despite fishery closures (Hutchinson 2008; Mieszkowska et al. 2009). It should here be noted that even when changes occur through ecology alone, that is, changes in species composition and/or size distribution within a community, recovery may also not occur rapidly, if the system has entered an alternative stable state (e.g., Scheffer et al. 2001; Bundy and Fanning 2005; Persson et al. 2007). Having both demographic and evolutionary genetics information is therefore of high importance for understanding contemporary dynamics of populations and thereby for the conservation and sustainable management of harvested populations (Kuparinen and Merilä 2007; Palkovacs et al. 2013).

While knowledge of within-population distributions of genetic and phenotypic variation may help predict local population dynamics, their effects on ecosystems, and the future evolvability of populations, knowledge on between-population (*β*) diversity in harvested species (or complexes of closely related undescribed species) has been shown to help predict the stability of ecosystem services through the portfolio effect, that is, where genes, populations, and species are considered as assets similar to financial assets, where diversity of assets ensures stability (Figge 2004). For example, Schindler et al. (2010) concluded that if the Bristol Bay sockeye salmon (*Oncorhynchus nerka*, Fig.1B) consisted of a single population rather than the extant several hundred discrete populations, year to year fluctuations in numbers of returning salmon would be more than twice as high, resulting in ten times more frequent fisheries closures. Based on this, the authors concluded that the reliability of ecosystem services will erode with the sequential extinction of individual populations, long before the entire species is extinct (Schindler et al. 2010). To recognize the functional basis of such gradual loss of ecosystem services, and to prevent it, alpha, beta, and gamma diversity have to be assessed, monitored, and protected not just above, but also below the species level.

Such and related realizations lead us to conclude that an evolutionary approach is indispensable in many conservation situations. First, even relatively old biological diversity often escapes the eye of all but the specialized taxonomist, and detecting and characterizing such (‘cryptic’) diversity often requires evolutionary genetics approaches. Importantly, upon closer inspection, such ‘cryptic’ species more often than not turn out to be ecologically and phenotypically differentiated (Bickford et al. 2007). Second, much ecologically relevant biological variation resides between and even within closely related populations and species that cannot be detected by traditional genetics based on few markers (such as barcoding), and detecting and robustly characterizing such variation requires an integrative evolutionary biology approach. Third, evolution often occurs on contemporary timescales and may irreversibly change the composition of a population or a set of populations in response to environmental change (Stockwell et al. 2003). In the following, we discuss several examples, where recent change in biodiversity can only be understood in light of evolutionary processes.

## The most common problems with ignoring evolution in monitoring

### Issues with ignoring evolutionary history and population differentiation

Threatened species that are composed of highly differentiated yet rapidly declining are a challenge to conservation management. For such species, it is crucial to understand the current population structure and historical relationships among populations, as well as the extent of adaptive variation within and adaptive differentiation between populations. Rheophilic fish, that is, fish with a preference for flowing water, include some of the most heavily managed fish populations in the world, and wild populations of rheophilic fish at the same time decline rapidly throughout Europe and North America (e.g., Aarts et al. 2004; Jelks et al. 2008; Limburg and Waldman 2009). Strong genetic differentiation is common among populations of rheophilic fish because rivers can be strongly isolated from each other, providing opportunity for high intraspecific between-population diversity. Appropriate decisions regarding conservation priorities and measures, including supportive stocking, will crucially depend on knowledge of all the above-mentioned variables.

Population diversity within species and between closely related species is an important genetic insurance for future environmental change, and it is often underappreciated by management and evolutionary ecologists alike that also currently, neutral genetic diversity that has built through longer periods of evolution in geographical isolation may become important for adaptation in the future (Paaby and Rockman 2014). Loss of such diversity through genetic homogenization, driven by the combination of heavy reliance on stocking and widespread ignorance of intraspecific diversity, is indeed widespread, affects species of high conservation priority (e.g., Muñoz-Fuentes et al. 2007; Keller et al. 2012; Gratton et al. 2014; Hudson et al. 2014) and has more generally been suggested to be one of the largest threats to freshwater fish diversity (Perry et al. 2002; Olden et al. 2004). While this problem has been reviewed for fish and other aquatic fauna in North America (Perry et al. 2002), it has received far less attention for fish in Europe, and for terrestrial organisms in general (see however Muñoz-Fuentes et al. 2007). In the following, we discuss an example where much, perhaps, most diversity has already been lost due to inadequate management: the species complex of European trout.

Trout are widespread in Europe north and south of the Alps. The evolutionary diversity of trout in Europe is reasonably well documented (Bernatchez 2001; Kottelat and Freyhof 2007), and we focus here on the Alpine region where many distinct lineages can be found in close geographical proximity, sometimes even in the same river (e.g., Giuffra et al. 1996; Gratton et al. 2014). Although locally declining in many places, trout can be found in almost every stream on either side of the Alps. However, this conceals the fact that most of the distinct trout species that occurred in different drainages of the Alps (Rhine-Atlantic *Salmo trutta* (Fig.1D), Rhone-Mediterranean *S. rhodanensis* (Fig.1E), Danubian *S. labrax*, Adriatic *S. cenerinus* and *S. marmoratus*) have been nearly entirely lost (Keller et al. 2011). Whereas the Atlantic trout (*S. trutta*) is very widespread and abundant in most of its range and now also in the ranges of all other species, all of the others have been impacted massively by genetic or ecological displacement, or both (Baric et al. 2010; Meraner et al. 2010, 2013; Keller et al. 2011). All of these are critically endangered, although conservation status is generally given only to *S. marmoratus* because the others rarely are recognized as distinct species by management (e.g., Kirchhofer et al. 2007).

The five described river trout species from Alpine drainages correspond to five evolutionary lineages all 0.2–2 million years divergent from one another (Bernatchez 2001; Gratton et al. 2014), and losing the diversity in this species complex through uncontrolled stocking or misguided management amounts to a cumulative loss of several million years of evolutionary history in just a few generations, and a very significant loss of both currently adaptive and currently cryptic genetic diversity. Stocking of trout has been carried out in Alpine streams and rivers for several centuries (Lorenz 1898). Stocking of millions of non-native Atlantic trout from hatcheries into Mediterranean, Adriatic, and Danubian watersheds every year has been the common place in the second half of the 20th century (Largiadèr and Scholl 1995; Mezzera and Largiadér 2001; Caudron et al. 2011; Keller et al. 2011). It has led to replacement of southern trout species in most streams and especially in the most heavily habitat-modified streams, whereas persistence with co-existence of species has been shown in a few locations with near-natural habitat structure (Baric et al. 2010; Meraner et al. 2010; Keller et al. 2012).

However, additional genetic differentiation can have evolved within lineages at much shorter time scales than those associated with the divergence between these old trout lineages, particularly when driven by recent or ongoing divergent selection between environments. Accordingly, trout display much diversity also within the distribution ranges of the ancient lineages, but this is only beginning to be discovered amidst a rapid rate of man-driven population homogenization. Gratton et al. (2014) showed evidence for speciation between populations of Italian trout belonging to the Adriatic lineage. Keller et al. (2012) showed evidence for parallel genetic adaptation along altitudinal habitat gradients within lineages in several Alpine rivers. The very different temperature and seasonality regimes between these habitats are likely to cause divergent selection on several different traits including immune system, egg-development rates, and juvenile growth rates (Robinson et al. 2010). Finally, there is evidence for local variation in trout morphology among streams at identical altitude but with different slopes, although the genetic basis of this is yet to be demonstrated (Stelkens et al. 2012).

To forestall further erosion of biodiversity in this key group of river fish, monitoring schemes for trout should urgently adopt an integrated perspective and collect individual and population level genetic and phenotypic data. Given the rapid advances in next-generation sequencing (NGS) techniques, this is a field where ecological genomics could make important contributions to genetic monitoring, to a better understanding of the scale and ecological basis of adaptation (Richardson et al. 2014), and to an evolutionarily informed management.

We chose to illustrate this section of our paper with the case of trout because it is a widespread and often abundant taxon of large ecological importance, is of major concern to fisheries and river management and restoration, and is probably more often central to ecological monitoring schemes than any other vertebrate animal, and the data situation is better than for most other species. That so much diversity has nevertheless been lost in trout due to misguided management and that also the current management leaves very much to wish for with regard to biodiversity conservation should therefore be taken as a severe warning. Trout have strong dispersal abilities. Therefore, we should expect to find at least as strong genetic population structure in many other aquatic taxa too that most often have weaker dispersal abilities, an expectation that is indeed supported by several recent publications on other rheophilic fish in the region (Nolte et al. 2009; Hudson et al. 2014). Unfortunately, this discovery comes amidst the realization of high rates of diversity loss (Persat 1996; Koskinen et al. 2002b; Duftner et al. 2005). Because fish are often able to hybridize for very many millions of years postspeciation (Scribner et al. 2000; Mendelson 2003; Bolnick and Near 2005; Stelkens et al. 2009), the problem of genetic displacement by misguided management is not expected to stop at the species boundary, and also here, the trout are no longer the only example. Work on European barbel species (Fig.1F) has demonstrated strong genetic displacement of Italian barbel (*Barbus plebejus*) by northern European barbel (*Barbus barbus*) driven by strong stocking propagule pressure within the range of the Italian species (Meraner et al. 2013), and northern roach (*Rutilus rutilus*, Fig.1G) is replacing southern endemics *Rutilus pigus* and *R. aula* in lakes in southern Switzerland (O. Seehausen pers. obs.). We are afraid that what has caught the attention of researchers is just the tip of the iceberg and that similar homogenization of intraspecific diversity is widespread across much of the heavily managed freshwaters of Europe and beyond. The same is likely to be true for managed populations of many terrestrial taxa, both animals and plants. While the negative consequences of loss of adaptive diversity are relatively easy to comprehend, we are nowhere near to be able to predict the long-term ecological and evolutionary consequences of the much larger loss of genetic variation that is currently ‘cryptic’ (Paaby and Rockman 2014). We believe that any credible biodiversity monitoring programs must take this problem seriously and must become equipped to measuring and detecting changes in genetic between- and within-population diversity.

### The problem of the within-site population homogeneity assumption

Management and monitoring of biodiversity very often builds on the premise that populations are genetically homogeneous within a given site. This is partly based on the assumption that current taxonomy has delimited species correctly. However, alpha taxonomy is insufficiently developed to justify this assumption for many taxa in many parts of the world, and this includes regions that are supposedly well known. The ‘within-site homogeneity’ paradigm also has a strong parallel in evolutionary biology, where it was long thought that gene flow would make population divergence at small spatial scale nearly impossible and maintenance of genetical distinctiveness in secondary contact of closely related populations difficult (Mayr 1942). However, there is increasing evidence that populations in secondary contact can remain differentiated in sympatry and that speciation may happen in the face of gene flow. This is best illustrated by the growing literature on parapatric and sympatric speciation (Bolnick and Fitzpatrick 2007; Richardson et al. 2014; Seehausen et al. 2014). However, these recent developments in speciation research have yet to leave their mark on applied biodiversity sciences. In the following sections, we will illustrate this with two very different examples. Our first example (this section) illustrates cryptic incipient species structure due to secondary contact within geographically defined populations of a taxon of management and conservation concern. The second example (next section) illustrates cases of sympatric origination of phenotypic life-history polymorphisms.

Grayling (*Thymallus thymallus*, Fig.1H) experiences widespread population decline across central Europe (e.g., Persat 1996; Uiblein et al. 2000; Koskinen et al. 2001, 2002a). Early analyses based on allozymes showed deep evolutionary divergence between grayling populations from some of the major river systems of Europe. The headwaters of several of these river systems can be near to each other in the Alps (Eppe and Persat 1999). More recent work using microsatellite DNA plus mitochondrial sequences showed deep population structure even within the Swiss Rhine basin (Vonlanthen et al. 2010). These studies not only revealed genetic distinctiveness of populations from different Rhine tributaries, but also found sympatric co-existence of genetically distinct populations within rivers, suggesting at least partial reproductive isolation after secondary contact. Current management of grayling populations includes habitat restoration and supplementary stocking, but thorough assessment of within-river population structure is clearly needed. The finding of sympatrically occurring distinct genotypic clusters in grayling is paralleled by sympatric occurrence of distinct genetic types of sculpins (*Cottus gobio*, Fig.1I) in some Swiss rivers based on analyses of AFLPs and microsatellite DNA (Hellmann 2011; Junker et al. 2012). However, these sympatric occurrences of genetically distinct types of rheophilic fish in Alpine streams are only a few examples of the increased number of observations of genetically distinct populations of the same taxonomic ‘species’, living in sympatry. While quite some discussion in evolutionary biology has focused on the origin of genetically distinct populations in sympatry (albeit rarely so in riverine fish), very little focus has been devoted in applied circles to how such sympatric forms can be recognized and how they should be managed.

It is clear that biodiversity monitoring needs to actively embrace the shifting paradigms in evolutionary biology in order to systematically look for and document sympatric populations, including old cryptic species (e.g., Bickford et al. 2007) and young species that have arisen by ecological speciation in response to ecological opportunity (Rundle and Nosil 2005; Schluter 2009). However, to be able to do so, collaboration of evolutionary biologists, taxonomists, conservationists, and managers is needed. Together, research and application should develop a conceptual and methodological framework that enables systematic recognition of sympatric species diversity within groups of closely related taxa. Importantly, this will require the simultaneous assessment of individual variation in phenotype and multilocus genotype. We wish to emphasize that this approach is distinct from genetic barcoding. The latter, which typically relies on the sequencing of a stretch of mitochondrial DNA, such as COI, works for old and allopatric divergence, where mitochondrial sequences became sorted between populations due to genetic drift in the absence of gene flow. When time was insufficient or gene flow has occurred, the sequence diversity visible to barcoding is unrelated to the diversity of species as illustrated by the diverse radiation of endemic whitefish (Fig.1J) in Alpine lakes (Hudson et al. 2011). Barcoding would estimate two old taxa in this radiation, but in fact these are ancient gene lineages that no longer represent different species, whereas more than 30 young species have evolved from the merger of these old lineages (Hudson et al. 2011).

### The problem with the individual equivalence assumption

The convention on biological diversity considers three different levels of diversity, that is, ecosystem, species, and genetic diversity (e.g., Laikre et al. 2009). However, intraspecific diversity on the phenotypic level, for example, morphological, physiological, behavioral, or life-history diversity, is ubiquitous in nature and central for contemporary evolution and ecosystem function. These different levels of phenotypic variation may often be linked, especially based on life-history variation, which often receives special focus in conservation and management. However, recognition of life-history variation within a single genetic population, such as in partial migration, will rarely be possible by monitoring of genotype or phenotype frequencies, but requires knowledge about the potential alternative life histories within the species. When these are relatively well understood, the relative frequency of the alternative life histories can potentially be monitored through phenotypic proxies of life history. However, in some cases, this may be less straightforward. Take the example of salmonid fish, classical examples of sympatric life-history polymorphism, notably involving resident and migratory forms. Whereas a part of the individual trajectory into a migratory or resident life history is determined by the environment (e.g., Olsson et al. 2006), a large part appears to be determined by underlying genotype (e.g., Jonsson 1982; Elliott 1989; Nichols et al. 2008; Hecht et al. 2013). If environmental change, such as loss of migratory connectivity, causes selection against the alleles that predispose individuals to be migratory, contemporary evolution would be expected to lead to a loss of the genetic predisposition for the migratory life-history form. However, as resident and migratory individuals are born in the same place and can most often not be phenotypically distinguished until shortly before onset of migration, even detailed monitoring programs paying attention to genetic and phenotypic variation may not sufficiently detect the presence of the distinct life-history forms. As salmonids are often keystone species (e.g., Willson and Halupka 1995), a change in migration pattern can potentially affect ecosystem dynamics, as seen in other species (Post et al. 2008; Brodersen et al. 2011; Bauer and Hoye 2014), and as migratory phenotypes are highly valued by recreational and commercial fishermen, such loss is likely to have substantial ecological and economic consequences. More subtle variation in the relative abundance of the two different life-history forms can lead to variation in size structure and seasonality of density among populations. Size structure and density are variables classically assessed in ecological monitoring, but to interpret data on these in species that may or may not contain migratory life-history variation, it is necessary to know about life-history distribution in the population. Low abundance of adults can be the result of high mortality or of migration. Where the former obviously could be critical for the population and would call for a change in management, the latter could be the desired scenario. It may thus be important to monitor variation in the frequency of different life-history forms for appropriate interpretation of population status and management of ecological diversity.

Similarly to the potential phenotypic variation in life history described above, individuals within a population may display distinct individual foraging strategies (e.g., Bolnick et al. 2003), behavioral syndromes (Sih et al. 2004), morphologies (e.g., Svanbäck and Eklöv 2003), or physiologies (e.g., Hoar 1976). Distinct behavioral types, for example, bold versus shy, can often be determined with relatively simple standardized trials (e.g., Chapman et al. 2011), albeit this may be difficult to implement in many monitoring programs. However, distinct physiology can often be analyzed by standardized tissue analyses (e.g., Boel et al. 2014), morphology by relatively simple geometric morphometric analyses (Zelditch et al. 2012), and individual ecology by stable isotope analyses (Post 2002). This further exemplifies the need for carefully collecting and storing material postsampling.

## The importance of historical collections

Ongoing evolutionary process can sometimes be inferred from genetic analyses of contemporary samples. This is, however, more difficult for evolutionary changes in the genetic composition of populations that occurred over decades and impossible for changes in phenotypic composition. Here, well-curated specimen and tissue collections have repeatedly been shown to be of great value for detecting and documenting contemporary phenotypic (Suarez and Tsutsui 2004; Carroll et al. 2005; Kitano et al. 2008) and genetic changes (Wandeler et al. 2007). Whitefish (*Coregonus* spp., Fig.1J) are one of the most extensively diversified fish in large and deep lakes of the Northern Hemisphere (Turgeon and Bernatchez 2003; Hudson et al. 2011). In the archipelago of large and deep pre-Alpine lakes on the north slope of the European Alps, they have radiated into more than 30 distinct species since the retreat of the glaciers (Hudson et al. 2011). However, much of this diversity has been lost rapidly in the past few decades as a consequence of lake eutrophication (Vonlanthen et al. 2012). Based on genetic analyses of DNA extracted from a collection of historical scales maintained at the Institute for Lake Research and Fisheries Langenargen (Baden Württemberg, Germany) that were collected before and during eutrophication and from contemporary samples of the re-oligotrophication phase, it was shown that the process leading to this loss of species richness was speciation reversal rather than classical extinction (Vonlanthen et al. 2012), driven by the loss of deep water habitat and displacement of deep water species to shallower depths due to oxygen depletion of deep water and sediments. Importantly, the historical scale collection was so valuable in this case only because at the Langenargen institute, whitefish samples had always been identified to species level, something that was rarely done in other whitefish lakes.

Similarly, only through comparison with well-annotated historical collections did it become apparent that the major ecosystem perturbations in East Africa's Lake Victoria were associated not just with the sudden loss of several hundred species of endemic cichlid fish (Fig.1K), but that many of the surviving species were undergoing major evolutionary changes, most likely due to the interaction of increased interspecific hybridization with changed selection pressures (Seehausen et al. 1997; Witte et al. 2013). The documentation of the recent collapse of a sympatric species pair of stickleback (Fig.1L) in Enos Lake, Canada, and that of a whitefish species pair in Lake Skrukkebukta, Norway, back into a single admixed population too became possible only through quantitative phenotypic comparison of older collections and new ones (Taylor et al. 2006; Bhat et al. 2014). Importantly, there are many other cases around the world where written and oral reports suggest major loss of species diversity has occurred due to human impacts on ecosystems, but in most cases, the evidence remains anecdotal because of the absence of historical collections (Seehausen 2006).

Apart from documenting the loss of diversity, access to historical samples can be the key to successful management of local populations. One such example can be found in the nine Danish Atlantic salmon (*Salmo salar*) streams, which originally each contained a genetically distinct population of Atlantic salmon (Geertz-Hansen and Jørgensen 1996). Salmon in Danish streams were until recently managed through stocking of offspring from foreign stocks, and original stocks were generally assumed to be extinct (Geertz-Hansen and Jørgensen 1996). However, based on analyses of DNA extracted from 60- to 80-year-old scale samples and from contemporary individuals, Nielsen et al. (1997, 1999, 2001) found that original stocks still occurred among introduced stocks in three rivers. This led to an immediate change of management strategy, where parental fish were exclusively collected locally from the river and genotypically assigned before being used in the breeding program. As a result of this, local indigenous populations have now recovered considerably, where they were still found (Nielsen and Hansen 2008).

Historical samples were in all the above examples absolutely necessary to detect, measure, and understand contemporary changes in biodiversity through evolutionary processes. For management at species level, baselines on what is natural may be shifting when relying on contemporary data (Pauly 1995; Baum and Myers 2004; Knowlton and Jackson 2008). Similarly, baseline diversity assessments have to be implemented on genetic and phenotypic level in order to combat the shifting baseline syndrome in management of biodiversity. This is ideally combined with historical DNA analyses to attempt to correct the already shifted baselines. It should here be further emphasized that contemporary stored samples with time will become highly valued historic samples. Monitoring programs should therefore not only target description of present state but also take the extra effort to build collections of reference samples that will no doubt become of immense value for managers and scientists alike within just a few years.

## Large-scale assessment of ecosystems and biodiversity

### Missed opportunities in ecological monitoring

Numerous major efforts are being made worldwide to monitor the structure and dynamics of biodiversity in both terrestrial and aquatic ecosystems. For example, the US National Science Foundation awarded major funding for the construction of a National Ecological Observatory Network (NEON), with a construction phase expected to last 5–7 years, and full operation to begin in 2016 or later. NEON will be the first observatory designed to detect and enable forecasting of ecological change at continental scales over multiple decades. Its vision is to guide understanding and decisions in a changing environment with scientific information about continental-scale ecology through integrated observations, experiments, and forecasts (http://www.neoninc.org/).

More generally, bird populations are monitored throughout the world through netting, ringing, and visual observation, marine fish populations are monitored worldwide through underwater visual census, test fishing, and evaluations of commercial catches, and freshwater fish communities are monitored intensively in Europe and North America using standardized electrofishing and gill netting. Ecological monitoring is widespread and relatively well developed to the extent that some important ecological trends can be detected and the driving processes identified. For example, the European standardized monitoring of lake fish assemblages through standardized gillnet survey fishing (Comité Européen de Normalisation 2005) has been crucial for our understanding of the ecological role of fish in shallow lake ecosystems (e.g., Jeppesen et al. 2000, 2005; Mehner 2010).

The data from large-scale ecological surveys are in some cases located in public databases. An example of this is the Swedish NORS database, which contains survey fishing data from more than 3000 lakes, 28 of which on at least 20 different occasions, starting from more than half a century ago (Kinnerbäck 2013). This database has led to a number of analyses furthering our understanding of the ecological role and success of different fish species in lakes and how this has changed over time (e.g., Nyberg et al. 2001). However, based on the current monitoring design, only data on species composition, abundance, habitat association, length distribution, length–weight relationship, and in some cases length at age are collected and stored in the database. Generally, samples are not stored and taxonomic, phenotypic, and genetic data are not collected. If tissue and phenotypic samples had been taken and stored, for example, in form of standardized photographs or preserved specimens, it would now be possible to identify expected evolutionary responses to changing environments over half a century in parallel in multiple lakes and populations. Further, together with the quantitative survey data on abundance of different species, it would have been possible to gain profound understanding of the relative rates of ecological, demographic, and evolutionary responses to different aspects of environmental change, and about the interplay between ecological, evolutionary, and demographic processes. In turn, the database could now be a great resource for ecologists and evolutionary biologists alike and could contribute to the newest synthesis in ecology and evolution (Schoener 2011).

Whereas ecological monitoring is often part of governmental management (but see NEON as an exception), investigation of genetic diversity unfortunately mostly still relies on researcher-driven science projects (Schwartz et al. 2007). For example, Laikre et al. (2008) listed 775 molecular genetic studies of natural Swedish populations. However, these were generally carried out uncoupled from ecological monitoring programs, and as a consequence, both types of data are plagued with problems when researchers or managers would like to scale up in space and environmental context (a problem for many researcher-driven evolutionary projects) or in time (a problem for governmental monitoring programs).

### Needed development in monitoring concepts

If biological monitoring programs want to detect, quantify, and understand how evolutionary processes and ecological and demographic dynamics together determine changes in biological diversity in response to environmental change, they need to integrate classical ecological data, for example, abundance and distribution of classically recognized species, with data typically collected only in evolutionary studies, such as genetic, phenotypic, and functional variation and distinctiveness within and between populations, cryptic species, ecological species, ecotypes, and life-history variants. This recognition is not completely new, and it is reflected to variable degree in several recently launched international initiatives, such as NEON, the initiative of the Genomic Observatory Network, the Genetic Monitoring group, and the Group on Earth Observations Biodiversity Observation Network. We see this development as very positive, but raise concern about mistaking the barcode of life approach for assessment of evolutionary and genomic diversity. We stress the importance of paying attention to the level of biological integration between DNA sequence diversity and taxonomically recognized species diversity and more specifically to the many species and divergently adapted populations that are invisible to barcoding but make up for a large wealth of biological diversity. If ‘genetic’ monitoring was reduced to barcoding, it would likely fail in describing biodiversity just as much as purely ‘ecological’ monitoring does.

We wish to emphasize that genetic, phenotypic, and ecological data have to be fully integrated in order to monitor variation in multilocus genotypes, cryptic populations, phenotypes, and ecotypes besides the monitoring of classically recognized species. This will require an increased amount of postsampling analytical work, but the approach can, for instance, be implemented by initially focusing on key taxa while storing the samples and their annotations of all other taxa for possible future work. Such focal taxa are referred to as sentinel taxa in the planning of NEON (Schimel et al. 2011). How these focal taxa are chosen will to some extent depend on the primary goals of the monitoring. In many cases, it may be useful to have some taxa of key conservation concern and others that play key roles in the ecosystem. We suggest sampling protocols in existing monitoring programs be adapted, standardized postsampling analytical protocols be developed for sentinel taxa, and tissue samples of sentinel and other taxa be preserved for future work. The kind of genetic and phenotypic data to be collected will have to be chosen in each program according to their relevance for monitoring and scientific investigation. For allowing broad-scale and long-term comparisons, we emphasize the importance of standardized methods, appropriate sample sizes, standardized data management, and open access data sharing.

## Suggested future monitoring strategies

### Examples of implementation of evolution-aware monitoring

Given that several of the large international monitoring programs are still in the planning and development phase, we chose here to report from two much smaller programs for which we sign responsible. We have during the last 5 years developed and implemented two monitoring programs that quantitatively assess biodiversity of fish in pre- and subalpine lakes (Projet Lac) and rivers (Progetto Fiumi) of the European Alps and explicitly incorporate evolutionary process. The motivation for launching such monitoring programs derived from our history of discovering taxonomically unrecognized and underappreciated diversity of endemic species of fish in these and other systems, and the observation that such diversity was generally being lost at unprecedented rates wherever ecosystems got perturbed (reviewed in Seehausen 2006; Vonlanthen et al. 2012).

Projet Lac (http://www.eawag.ch/forschung/fishec/gruppen/lac/index_EN) and Progetto Fiumi were designed to quantitatively assess fish diversity from genes and phenotypes within populations to alpha, beta, and gamma species diversity including old and taxonomically recognized as well as young adaptive radiation species in pre-alpine and subalpine lakes and rivers, respectively, of the European Alps. In Progetto Fiumi, monitoring is conducted with a combination of quantitative and qualitative sampling and recording of individual genetic, phenotypic, and ecological variables. The combination of quantitative and qualitative sampling allows obtaining information on the community and population structure (quantitative sampling) and also obtaining samples of rare species and phenotypes without having to process unnecessarily large numbers of the most abundant species and phenotypes (qualitative sampling). Projet Lac uses standardized fish population surveys, following the standardized European protocol (Comité Européen de Normalisation 2005) and a locally developed complementary protocol that pays special attention to deep sections of the lakes that are not well sampled by the European protocol (Alexander et al. 2014). During sampling, information on habitat variables is being recorded to enable later determination of phenotype–habitat associations.

Both projects follow European standards of quantitative survey fishing. However, whereas the European protocol discards the fish after crude taxonomic identification to species complex or super species (e.g., ‘trout’ or ‘whitefish’), and measuring size (a subsample of fish are often used for length–weight relationship and age analysis), Projet Lac and Progetto Fiumi are preparing and storing rich information from many individual fish. The first step is measuring and weighing individual fish followed by the preparation of standardized photographs of freshly caught individuals for downstream taxonomic, color, and geometric morphometric analyses. Next, we collect tissue samples for genetic analysis of at least 30 individuals of the more common species from each lake and stream, but up to several hundred when a species occupies multiple distinct habitats (i.e., 30 individuals per lake habitat), and of all individuals of less abundant species. Finally, we individually label and preserve all individuals of rare species and at least 30 of every more abundant species as whole-preserved museum specimens. All individuals are individually labeled, with labels matching between specimen, photographs, tissue samples, and habitat variables, allowing for detailed analyses of individual variation and adaptation.

Projet Lac quickly discovered several major trans-alpine species invasions and range expansions that had gone unnoticed in standard fishery surveys, and recorded many previously undescribed, often phenotypically highly distinct populations some of which will likely prove to be new species. By now, Projet Lac has completed assessments of about 20 lakes, including some of the largest lakes of Europe, and begins to reveal major loss of endemic species and functional diversity of fish during lake eutrophication in several taxonomic groups.

### Suggested development of biodiversity monitoring

Building on our experience with the two projects described above, we suggest changes in the way biological samples be collected and processed in future monitoring programs. These changes concern choice of sites and habitats for sampling, sampling and subsampling design, processing protocol, and conservation of samples. The specifics of the design will depend on trade-offs regarding number of habitats or sites sampled, number of individuals sampled per site or habitat, and amount of data or material collected per sampled individual. All of this will also be quite different between surveys of aquatic communities, terrestrial animal communities, and plant communities. Our own experience lies with aquatic communities, but we think that many of the general considerations can be applied broadly.

Existing monitoring programs for aquatic ecosystems often allocate most if not all effort into habitats with the highest abundance of organisms. For example, in the European Lake Fish survey program, the deepest areas of a lake are not sampled at all, due to the expected low abundance of fish there (Comité Européen de Normalisation 2005). Yet, the deep benthic zone is precisely the area where the most ecologically distinct phenotypes and endemic species are expected (e.g., Kottelat and Freyhof 2007). Clearly, monitoring programs that care about biodiversity change must sample all habitats types, despite that some are likely to yield low catches or are less abundant (Alexander et al. 2014).

Regarding collection of samples, we stress the importance of the quantitative approach, which is the base of many of the existing monitoring concepts. This will enable a possibility to link evolutionary processes with ecological structure. However, to get, for example, rare phenotypes or species, semi-quantitative or qualitative methods may be an important supplement to the quantitative approach. As many communities are dominated by a few abundant species, it will most often be necessary with some degree of subsampling, when choosing individuals for subsequent genetic, morphological, or ecological analyses or tissue or whole body collection. In the choice of individuals, it is for obvious reasons important to record whether individuals have been chosen based on their uniqueness or as a random subsample. Both will have their merits in biodiversity surveys, but have to be distinguished. The number of individuals chosen for subsamples will often be context specific, where some techniques allow processing of a high number of individuals in little time.

We emphasize the value of sampling techniques that permit measuring the maximum number of potentially important traits while minimizing effort. For example, standardized photographs of individuals allow quantifying many external morphological traits. In addition, they are inexpensive, relatively fast, easily archived, and require only a moderate amount of training, all important attributes to allow easy integration into monitoring programs. Where many monitoring efforts require relatively large sampling effort, that is, measured either in number of people involved or in time spent per individual person involved, an additional person taking photographs of all sampled individual will often not add substantially to the cost of the survey program.

It is important that samples are not only stored for future researchers, but also processed, analyzed, and – most importantly – well annotated as a part of the monitoring program (see the point above about the historical scale collections of whitefish species in Lake Constance). Genetic information, for example, in the form of microsatellite data, for a single population may be of limited value. However, as more standardized data becomes available within a region, it will be possible to determine the distinctiveness of each population. NGS techniques now offer much better opportunities for discovering population structure, adaptation, selection, and species delimitation and for detecting changes in any of these as a consequence of environmental change (Larson et al. 2014; Wagner et al. 2014). While the generation and analyses of NGS sequence data may still be too complex to apply broadly at this stage, we strongly recommend samples should still be stored in such a way, for example, in pure ethanol or suitable buffer solutions, as to enable such genomic analyses in the future.

We also like to emphasize that there is a need to grow expertise in seeing and quantifying phenotypic variation in monitoring programs. Functionally and taxonomically relevant natural variation in phenotypes is not easy to detect by standardized analyses that are not optimized for each taxon separately. Its discovery and description used to be the unique skill of experienced naturalists, and today's biology students are rarely receiving such training. We think the only way to achieve this goal in the long term is by re-invigorating the training in field taxonomy in the ecology and evolution curricula.

## Concluding remarks

In conclusion, we see an urgent need to integrate evolutionary process into biodiversity monitoring programs. We also anticipate that this is most likely to come about through an increased dialog with mutual appreciation between conservation practice, nature management, and curiosity-driven ecologists and evolutionary biologists. This two-way process will be greatly facilitated once evolutionary biologists can take advantage of monitoring programs, and conservation practice can take advantage of the knowledge base and methods of evolutionary biologists to achieve a process-based monitoring and management of natural populations, communities, and ecosystems.
